# Assessing personalized molecular portraits underlying endothelial-to-mesenchymal transition within pulmonary arterial hypertension

**DOI:** 10.1186/s10020-024-00963-z

**Published:** 2024-10-26

**Authors:** Ruhao Wu, Ge Zhang, Mingzhou Guo, Yue Li, Lu Qin, Tianci Jiang, Pengfei Li, Yu Wang, Ke Wang, Yize Liu, Zhiqiu He, Zhe Cheng

**Affiliations:** 1 Department of Respiratory and Critical Care Medicine, The First Affiliated Hospital of Zhengzhou University, Zhengzhou, 450052 Henan China; 2 Department of Cardiology, First Affiliated Hospital of Zhengzhou University, Zhengzhou, 450052 Henan China; 3 Henan Province Clinical Research Center for Cardiovascular Diseases, Zhengzhou, Henan China; 4 Key Laboratory of Cardiac Injury and Repair of Henan Province, Zhengzhou, 450018 Henan China

**Keywords:** Endothelial-to-mesenchymal transition, Pulmonary arterial hypertension, Machine learning, Biomarker, Molecular signature

## Abstract

Pulmonary arterial hypertension (PAH) is a progressive and rapidly fatal disease with an intricate etiology. Identifying biomarkers for early PAH lesions based on the exploration of subtle biological processes is significant for timely diagnosis and treatment. In the present study, nine distinct cell populations identified based on gene expression profiles revealed high heterogeneity in cell composition ratio, biological function, distribution preference, and communication patterns in PAH. Notably, compared to other cells, endothelial cells (ECs) showed prominent variation in multiple perspectives. Further analysis demonstrated the endothelial-to-mesenchymal transition (EndMT) in ECs and identified a subgroup exhibiting a contrasting phenotype. Based on these findings, a machine-learning integrated program consisting of nine learners was developed to create a PAH Endothelial-to-mesenchymal transition Signature (PETS). This study identified cell populations underlying EndMT and furnished a potential tool that might be valuable for PAH diagnosis and new precise therapies.

**Supplementary Information:**

The online version contains supplementary material available at 10.1186/s10020-024-00963-z.

## Introduction

Pulmonary arterial hypertension (PAH) is a relatively uncommon yet severe vascular disorder marked by heightened blood pressure within the pulmonary artery (mean > 20 mm Hg) (Simonneau, et al. 2019; Mocumbi, et al. 2024), resulting in a progressive condition characterized by heightened strain on the right ventricle that may culminate in right heart failure and mortality (Humbert, et al. 2022). Timely diagnoses of PAH patients remain a research focus, with the aim of improving outcomes and providing more treatment options (Ruopp and Cockrill 2022).

The etiology of PAH is intricate, encompassing pulmonary vascular remodeling, augmented vasoconstriction, and inflammation in both arterial and microvascular structures (Dai, et al. 2018; Zhong and Yu 2022; Mocumbi, et al. 2024). Endothelial cells (ECs) play a crucial role in the initial vascular alterations that precipitate PAH, which involves in dysfunctional signaling, injury, and apoptosis (Evans, et al. 2021). Endothelial-to-mesenchymal transition (EndMT), in which ECs obtain characteristics of mesenchymal cells, is deemed to be involved in this pathogenic process (Ranchoux, et al. 2015; Xue, et al. 2020; Evans, et al. 2021). Following EndMT, there is an increase in EC proliferation and migration, leading to pulmonary vascular remodeling and the progression of PAH (Li, et al. 2018; Alvandi and Bischoff 2021).

Recently, high-throughput sequencing technology has advanced rapidly and is now commonly used in clinical settings, such as disease mechanism exploration and infection pathogen identification (Daniloski, et al. 2021; Zhang, et al. 2022; Li, et al. 2023; Ren, et al. 2024). It is also a novel and effective way to guide individualized treatment based on multi-omics information integration (Dave, et al. 2023). While numerous studies have been carried out using bulk RNA sequencing (RNA-seq) data to explore PAH in detail, limitations in resolution and cellular heterogeneity have hindered further exploration. However, single-cell RNA sequencing (scRNA-seq) technology has emerged as a robust platform for investigating alterations in cellular function, cell–cell communication, and cellular development at a more refined level (Aldridge and Teichmann 2020; Zhang et al. 2023a, b). Thus, single-cell technology is regarded as an ideal method for exploring cell heterogeneity and identifying specific pathogenic cell populations (Fernandez and Giannarelli 2022). For instance, Rodor and colleagues discovered endothelial cell function alterations in mice with PAH through the scRNA-seq platform (Rodor, et al. 2022). Besides, Ferrian et al. constructed the PAH single cell architectural map, identified unique inflammation cell subsets, and characterized them (Ferrian, et al. 2024). Nevertheless, at single-cell resolution, specific research remains largely deficient in PAH. Therefore, it is essential to undertake further scRNA-seq research to elucidate mechanisms and identify novel biomarkers in PAH.

Here, we systematically integrated both scRNA-seq and bulk RNA-seq data to depict biological characteristics of PAH patients. Further trajectory analyses were performed on ECs because of its apparent change in function and role in PAH. Based on the observed EndMT process and a unique EC subset, we obtained EndMT pattern genes (ETPGs). Afterward, we implemented a machine learning (ML) program integrating nine learners into our research to develop a PAH Endothelial-mesenchymal Transition Signature (PETS), which exhibited favorable performance in discriminating between PAH patients and healthy people.

## Materials and methods

### scRNA-seq data collecting, processing and analysis

The 10X single-cell transcriptome data from GSE169471 (including 31,444 cells) were downloaded from the Gene Expression Omnibus (GEO) (http://www.ncbi.nlm.nih.gov/geo/) database (Saygin, et al. 2020). Quality control of single-cell RNA sequencing data was executed with the following criteria: > 200 genes/cell, < 4000 genes/cell, > 3 cells/gene, and < 10% mitochondrial genes. Then, the integration work was conducted as follows: (i) For individual objects, perform Log-Normalization and identify 2000 highly variable genes (HVGs) using the “vst” method. (ii) Divide the combined object into a list based on the “SplitObject” function. (iii) Use the “FindIntegrationAnchors” function to identify the “anchors” in the different objects to construct a reference. (iv) Eliminate the batch effect of nine samples through the “IntegrateData” function. Finally, a Seurat object with a batch-corrected expression matrix for all cells was outputted (Butler, et al. 2018; Hao, et al. 2021). Depending on the expression of the top 2000 HVGs, the top 20 Principal Component Analysis (PCA) components were determined. Afterward, the first 20 significant PCs determined by the Elbow plot were incorporated to conduct cell clustering using the “FindNeighbors” function and “FindClusters” method (resolution = 0.5), which implements the Louvain network-based clustering algorithm. Subsequently, Uniform Manifold Approximation and Projection (UMAP) analysis was applied for further dimensional reduction and clustering visualization (Becht, et al. 2018). To annotate cell types, highly expressed genes of all cell subclusters were used as potential references and combined with canonical cell-type-specific surface markers derived from CellMarker (http://yikedaxue.slwshop.cn/index.php).

### Bulk data processing and analysis

The raw data were obtained from the GEO database, providing detailed information on the platform, samples, and GSE records. Probes were converted into gene symbols based on the annotation information from the platform. Subsequently, differential expression analysis was conducted using the limma package to identify differentially expressed genes (DEGs). Afterward, gene set enrichment analysis (GSEA) was performed using the clusterProfiler package.

### Tissue preference of distinct cell types

We calculated the observed-to-predicted cell number ratio (Ro/e) for each cell type in different groups to quantify the preference of each cell type (Zhang, et al. 2018). The chi-square test was used to determine the predicted cell numbers for each combination of cell types and groups.

### Assessing the contribution score of different cell types

In our study, the contribution score was calculated to assess the contribution of each cell cluster to PAH. The “FindAllMarkers” function was used to list the top 100 differentially expressed genes (DEGs) between two groups. Subsequently, we calculated the fold change in gene expression (FCexp) and the proportion of gene expression (FCprop) of PAH relative to the control group across different cell types. The square root of FCexp and FCprop was regarded as the contribution score.

### Functional enrichment analysis at single-cell resolution

Gene sets of immune, metabolism, signaling, and proliferation pathways were collected from MSigDB (Liberzon, et al. 2011). Then, the AUCell algorithm was utilized to assess the activity of various biological pathways in both the PAH and control groups (Aibar, et al. 2017). Pathway activity scores were compared between the two groups using empirical Bayes’ statistics.

### Cell–cell communication analysis

We applied Cellchat to depict cell–cell interactions among cell populations in both PAH and control groups (Jin, et al. 2021). Based on CellchatDB, a signaling molecule interaction database, over-expressed ligands and receptors were identified. Afterward, Cellchat quantifies intercellular communications by associating each interaction with a probability value, which is recalculated based on the average expression values of a ligand by one cell group and that of a receptor by another cell group, as well as their cofactors. We adopted the default settings of recommended pipelines to conduct a quantitative analysis of intercellular communications in an unsupervised manner.

### Pseudotime trajectory inference

After further dimensionality reduction and clustering, we utilized the Slingshot algorithm to reconstruct the differentiation trajectories of endothelial cells (Street, et al. 2018). The input for the Slingshot calculation and cell lineage trajectory formation consisted of UMAP embedding and subclustering assignments for 3165 endothelial cells from both PAH and control groups. Additionally, we applied the Monocle2 pseudotime algorithm to recapitulate the results (Qiu, et al. 2017). The “differentialGeneTest” function was employed to identify differentially expressed genes among clusters, followed by dimensionality reduction using the DDRTree method. Finally, cells were ordered and differentiation trajectories were built, while significant differentially expressed genes (qval < 0.1) were clustered to illustrate distinct expression patterns along the trajectory.

### Enrichment analysis

The “FindAllMarkers” function was used to list cell populations’ markers to further explore their functions. Next, enrichment analysis was conducted through the “RunEnrichment” function in the SCP package (https://github.com/zhanghao-njmu/SCP). Identified differentially expressed genes were subjected to Kyoto Encyclopedia of Genes and Genomes (KEGG) pathways and Gene Ontology (GO) terms enrichment analyses to depict functional differences along the pseudotime trajectory (Gene 2015; Kanehisa, et al. 2017). For the lung tissue RNA-sequencing cohort, gene set enrichment analysis (GSEA) was conducted to evaluate the biological pathway activities based on the ranked differential gene expression matrix (Subramanian, et al. 2005).

### Single-cell metabolic activity analysis

The assessment of metabolic activity at the single-cell level was conducted using scMetabolism, which utilized metabolic pathways from KEGG and Reactome entries to quantify metabolic activity through a single-cell matrix file and the Vision algorithm (Gillespie, et al. 2022; Wu, et al. 2022).

### Pivotal module genes identification

To identify pivotal genes associated with endothelial differentiation, high dimensional weighted gene co-expression network analysis (hdWGCNA) was executed as follows: (i) Construct an expression matrix from the single-cell object undergoing PCA and UMAP dimensional reduction; (ii) Based on the expression matrix, construct averaged “metacells” by grouping cells according to cell types; (iii) Normalize the metacell matrix and determine a suitable soft threshold β to create a topological overlap matrix (TOM); (iv) Establish the co-expression network and calculate the eigenvectors of the modules in order to identify module feature genes. The foregoing analyses were implemented based on hdWGCNA R package (Morabito, et al. 2021). Finally, the pivotal module and contained genes were recognized after evaluating the expression of module genes in distinct disease groups and endothelial subtypes.

Following the identification of pivotal module genes, EC3 significant altered genes were selected through FindAllMarkers function, applying a significance threshold of average log2 Fold Change > 1 and adjusted P value < 0.05. Their overlap genes were defined as the key genes involved in the EndMT process, called EndMT pattern genes (ETPGs).

### PETS generated from integrative machine-learning program

To explore a PAH endothelial-mesenchymal transition signature (PETS), we leveraged nine machine learning algorithms, comprising elastic-net-regularized generalized linear model (glmNet), bootstrap aggregation classification and regression trees (Bagged CART), Naive Bayes (NB), Partial Least Squares (pls), Backpropagation neural network (NNet), K-Nearest Neighbors (KNN), random forest (RF), boosted generalized linear model (glmBoost), classification and regression trees (CART) (Zhang, Cui, et al. 2023; Zhang, et al. 2024). The final signature was generated following such a pipeline: (i) The initial investigation of the signature was carried out in the cohort GSE117261, which was randomly split into discovery and testing cohorts at a ratio of 7:3. (ii) Nine machine learning algorithms were employed on ETPGs to individually fit models. In order to mitigate the risk of overfitting resulting from the excessive complexity of the model, a tenfold 10-repeated cross-validation approach was implemented to enhance the generalization capability of the discovery cohort. (iii) In the testing cohort, a consensus evaluation strategy was applied to assess effectiveness and applicability of all models, encompassing the accuracy, Harrell’s concordance index (C-index), F1-score, precision, recall, and the root mean square of the residuals (RMSR). The glmNet model was recognized as the optimal scheme.

### Cell lines and cultures

Primary human and mouse pulmonary artery endothelial cells (PAECs) were obtained from Wuhan Procell Life Technology company Wuhan Procell Life Science & Technology Corporation. All cells were cultured in Dulbecco's Modified Eagle Medium supplemented with 10% fetal bovine serum and maintained in a humidified incubator with 5% CO2 at 37 °C. The hypoxic PAECs were cultured in a 1% O2 incubator (Galaxy R; RS Bitotech, Alloa, UK) continually gassed with 5% CO2 and 90% N2.

### RNA extraction and real-time quantitative PCR

Total RNA was isolated using Trizol (Takara, Dalian, China) according to the manufacture’s instruction. RNA concentration was determined by Nanodrop analysis. cDNA was synthesized from total RNA using PrimeScript RT reagent kit (Takara, Dalian, China). Quantitative RT-PCR was performed using SYBR Green Mix (Takara, Dalian, China). The primer sequences were as follows: Rarres2 forward, 5′-TTGCTGATCTCCCTAGCCCTA-3′, reverse, 5′-TGGGTGTTTGTGGAACTCCTC-3′. Tc2n forward, 5′-CACCTGATTTAAGTAGGCGCTT-3′, reverse, 5′-GGATGAATCGCTGAGATGAAGTT-3′. RARRES2 forward, 5′-AGAAACCCGAGTGCAAAGTCA-3′, reverse, 5′-AGAACTTGGGTCTCTATGGGG-3′. TC2N forward, 5′-TGGCTGTACTGAGGATTATTTGC-3′, reverse, 5′-TGTGAAGGAGTTTCTTGTGTCC-3′. CBY1 forward, 5′-TCTTTGGGAATACGTTCAGTCCG-3′, reverse, 5′-CCAGGTTCATAGTCGGGGA-3′. CKS1B forward, 5′-TATTCGGACAAATACGACGACG-3′, reverse, 5′-CGCCAAGATTCCTCCATTCAGA-3′. MSRB3 forward, 5′-CGGTTCAGGTTGGCCTTCATT-3′, reverse, 5′-GTGCATCCCATAGGAAAAGTCA-3′. SMAGP forward, 5′-ACCAGCCTCCTGACTACTCC-3′, reverse, 5′-CTTGCCACAGGACACCTTCA-3′.

The cycling conditions were as follows: initial denaturation for 10 min at 95 °C followed by 40 cycles of denaturation (15 s at 95 °C), annealing and elongation (30 s at 60 °C). The relative expression of gene was calculated using 2^ − △△Ct^ method using β-Actin as the reference gene.

### Mouse hypoxic PAH model and immunofluorescence Staining

Adult male Sprague–Dawley C57BL/6 J mice were purchased from the Laboratory Animal Center, Tongji Medical College. The mouse were placed in a normobaric chamber (Oxycycler model A84XOV; BioSpherix, Lacona, NY), and the oxygen concentration was adjusted to 10% for 4 weeks for hypoxia treatment. Hemodynamic studies and right ventricular hypertrophy were evaluated as previously reported (Tang, et al. 2022). The lungs of the mice were fixed by 4% paraformaldehyde and routinely processed into paraffin sections, 4 mm in thickness, for immunostaining examination. Paraffin sections of the lung were stained with DAPI (4′,6-diamidino-2-phenylindole), anti-CD31 (1:100; Proteintech Group), and anti-chemerin (1:100; Proteintech Group).

### Statistical analysis

**Fig. 1:**
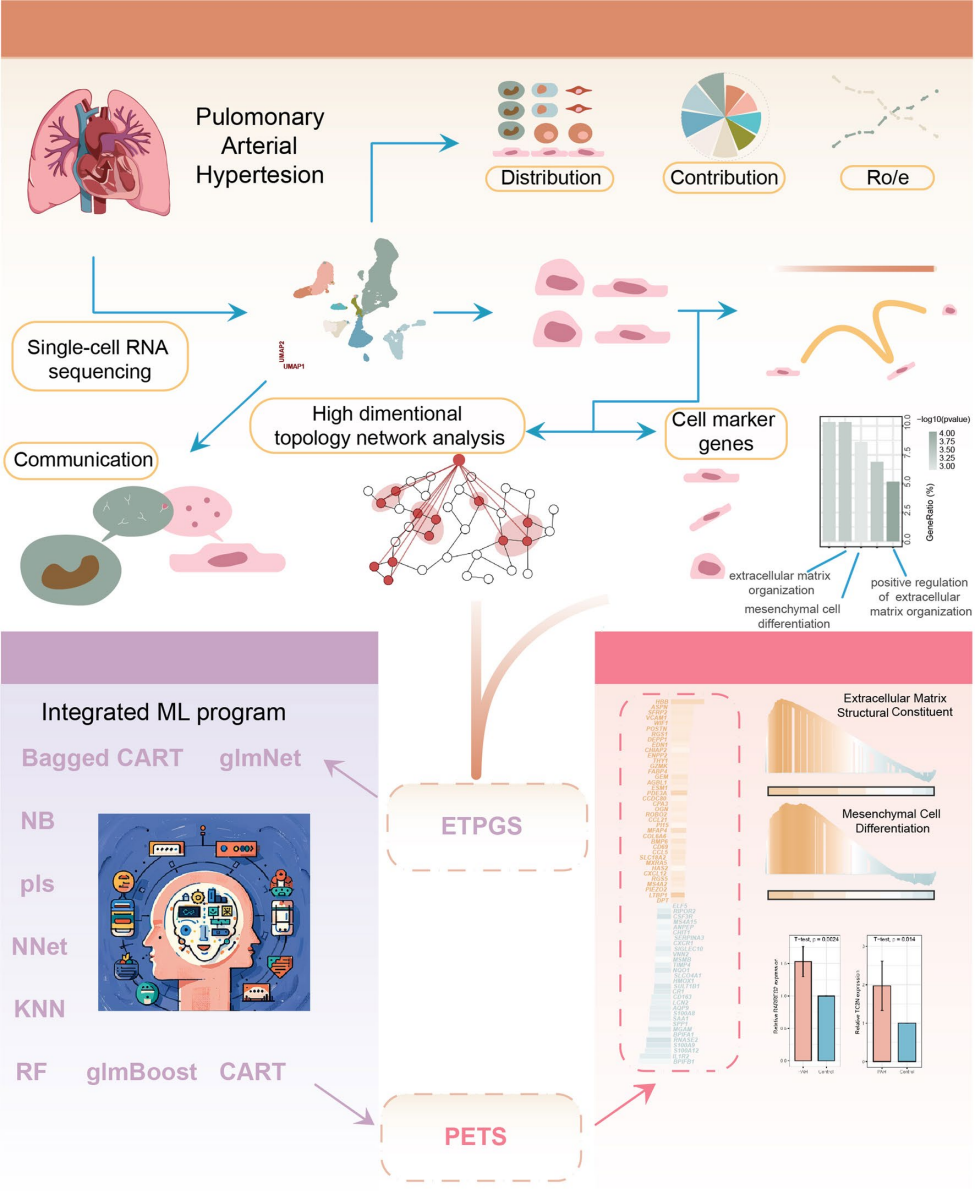
Flowchart illustrating the overall design of the study

All data processing, plotting, and statistical analysis were conducted in R (version 4.1.2). Kruskal–Wallis test was applied for oxidative phosphorylation level comparison among three EC subsets. The T test was applied to compare continuous variables. For all statistical tests, a two-sided P < 0.05 and FDR < 0.05 was deemed statistically significant (Fig. 1).

## Results

### scRNA‑seq analysis revealed cell landscape and characteristics in PAH

**Fig. 2:**
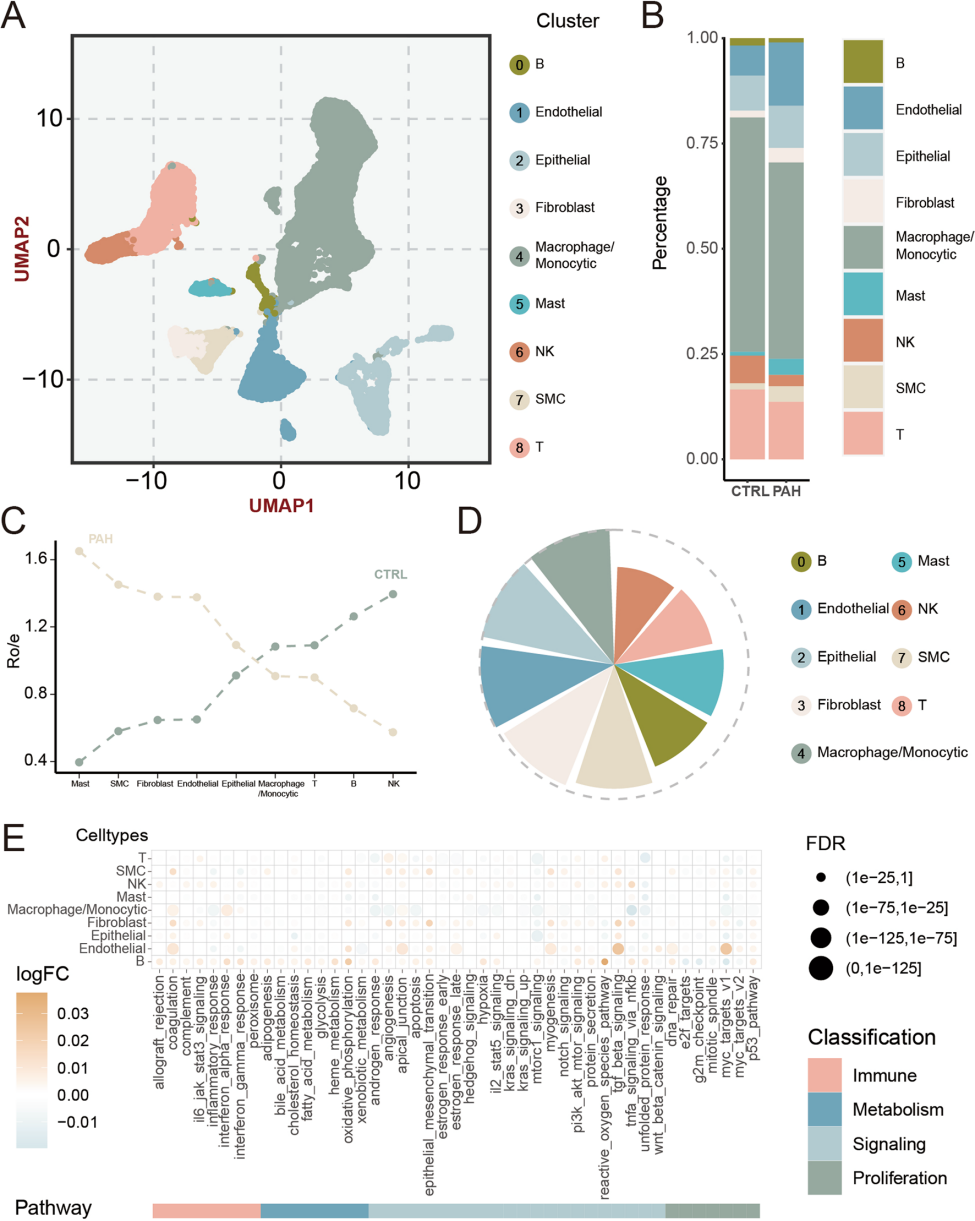
scRNA-seq depicted the cell landscape in PAH.** A** Cell landscapes of 28906 cells from 9 types (B cells, endothelial cells, epithelial cells, fibroblasts, macrophages/monocytes, mast cells, natural killer cells, smooth muscle cells, and T cells). **B** Cell proportion in PAH and control groups. **C** Line chart showing tissue prevalence for each cell type estimated by Ro/e score. **D** Pie chart exhibiting contribution score of distinct cell types.** E** Dot plots showing differentially enriched pathways in the global cell types

The 31,444 cells from three PAH samples and six control samples were analyzed via 10X scRNA-seq. After quality control, 28,906 cells were ultimately retained (Figure S1). Subsequently, UMAP analysis revealed 21 cell clusters (Figure S2A), which were further annotated into the following cell types: B cells, endothelial cells (ECs), epithelial cells, fibroblasts, macrophages/monocytes, mast cells, natural killer (NK) cells, smooth muscle cells (SMCs), and T cells (Figure S2B, Fig. 2A). We then quantified PAH-driven alterations in the cell type proportions. As depicted in Fig. 2B and S3, there was an increase in ECs, SMCs, and fibroblasts in the PAH group.

To measure the tissue enrichment of cell populations, we carried out Ro/e analysis. Similarly, most immune cells preferred to distribute themselves in the normal lungs, whereas mast cells and stromal cells were preferentially located in the PAH lung tissues (Fig. 2C). Afterward, we assessed distinct cell populations’ contribution scores. For differences caused by PAH at the single-cell resolution, macrophages/monocytes and ECs accounted for significant contribution, while NK cells, T cells, and mast cells exhibited limited impact (Fig. 2D, Table S1, 2).

Immunology, metabolism, signaling, and proliferation hallmark gene sets were used to investigate major variations in pathways between the PAH and control group (Table S3). Notably, several specific pathways displayed significant enrichment in all stromal cells, such as coagulation, epithelial mesenchymal transition (EMT), apical junction, myogenesis, and TGF-β signaling pathway, implying the presence of potential shared biological processes in PAH (Fig. 2E). Of these, the most significant changes occurred in endothelial cells, which were more enriched in oxidative phosphorylation and MYC target V1 pathways.

### Cell–cell communications of cell populations

**Fig. 3:**
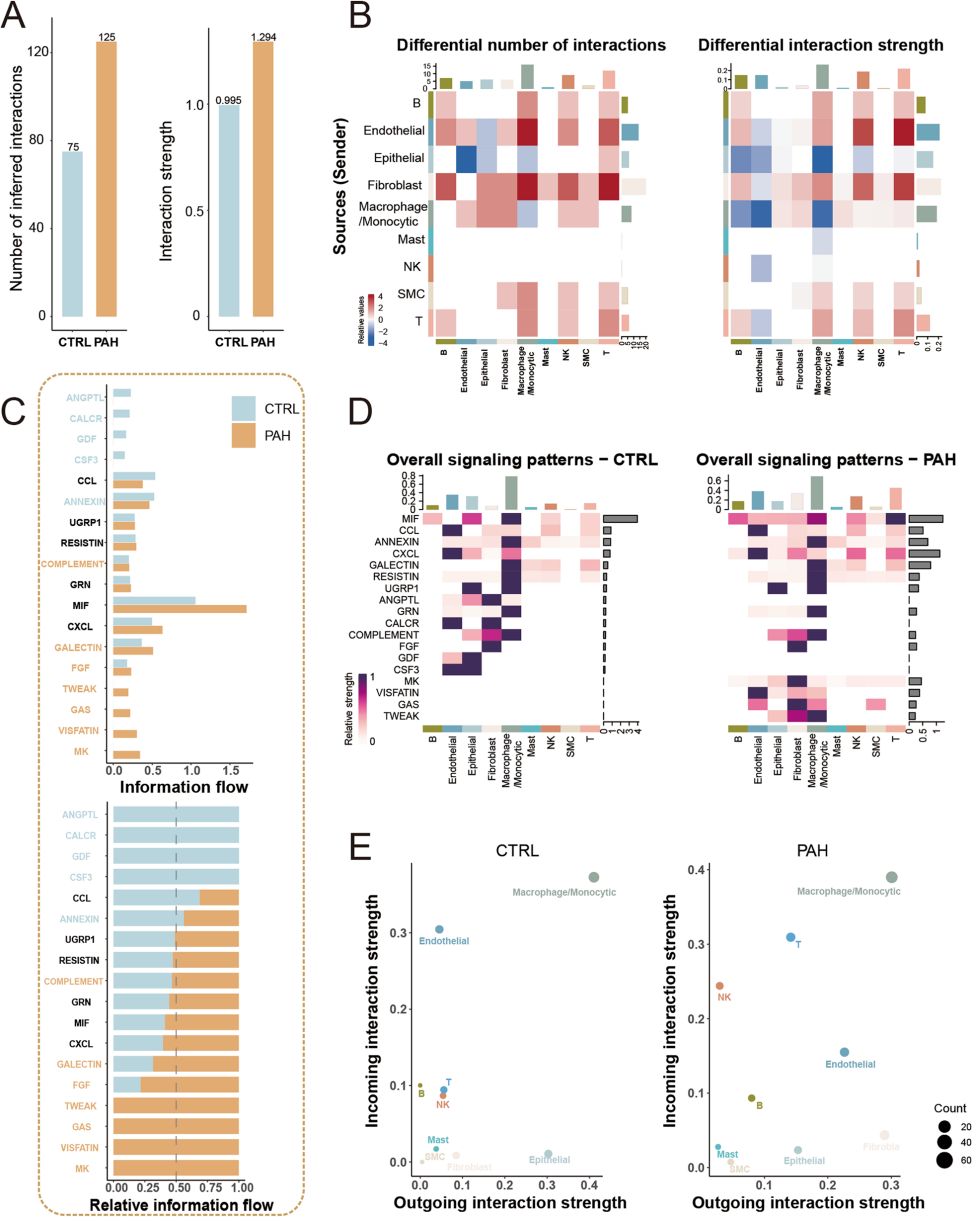
Cell–cell interaction differences in PAH. **A** Bar plot demonstrating elevated intercellular interaction number and strength in PAH. **B** Heatmap showing alterations in cell–cell interactions among distinct cell types. **C**-**D** Diverging activities between two groups and among specific cell types within each group in 18 enriched signaling pathways. **E** Variation of out-degree and in-degree of cell populations within the communication network in PAH

Relying on CellChat established ligand-receptor pairs and their cofactors, we constructed a cell–cell communication network (Figure S4A). Attractively, elevated intercellular interactions numbers and strength were detected in the PAH group (Fig. 3A). Remarkable enhanced interactions of ECs and fibroblasts with B cells, T cells, NK cells, and macrophages/monocytes probably accounted for it (Fig. 3B). Then, we identified 18 enriched signaling pathways based on intercellular interaction information. MK, VSFATIN, GAS, and TWEAK signaling pathways occupied a dominant position in the PAH group, while ANGPTL, CALCR, GDF, and CSF3 signaling pathways were specifically enriched in the control group (Fig. 3C). In regard to these pathways, a detailed analysis of changes in signaling-receptor levels revealed that PAH-dominant signaling pathways were induced by ECs, fibroblasts, and macrophages/monocytes (Fig. 3D). Interestingly, in the PAH group, ECs, epithelial cells, and fibroblasts exhibited significantly reduced activity in control-dominant signaling pathways (Fig. 3D).

Finally, we deduced the transformation of cell population roles based on changes in out-degree and in-degree within the communication network (Fig. 3E). In PAH, epithelial cells and fibroblasts displayed increased outgoing interactions, whereas NK cells and T cells exhibited higher incoming interactions. Notably, ECs possessed lower incoming interactions and higher outgoing interactions in the PAH group, which signified the change from the signaling sender to a signaling receiver. Overall, further investigation of ECs is pressing due to extensive and significant changes in comprehensive exploration containing cell proportion, Ro/e analysis, cell contribution, and cell–cell communication.

### Trajectory analyses revealed EndMT in endothelial populations

**Fig. 4:**
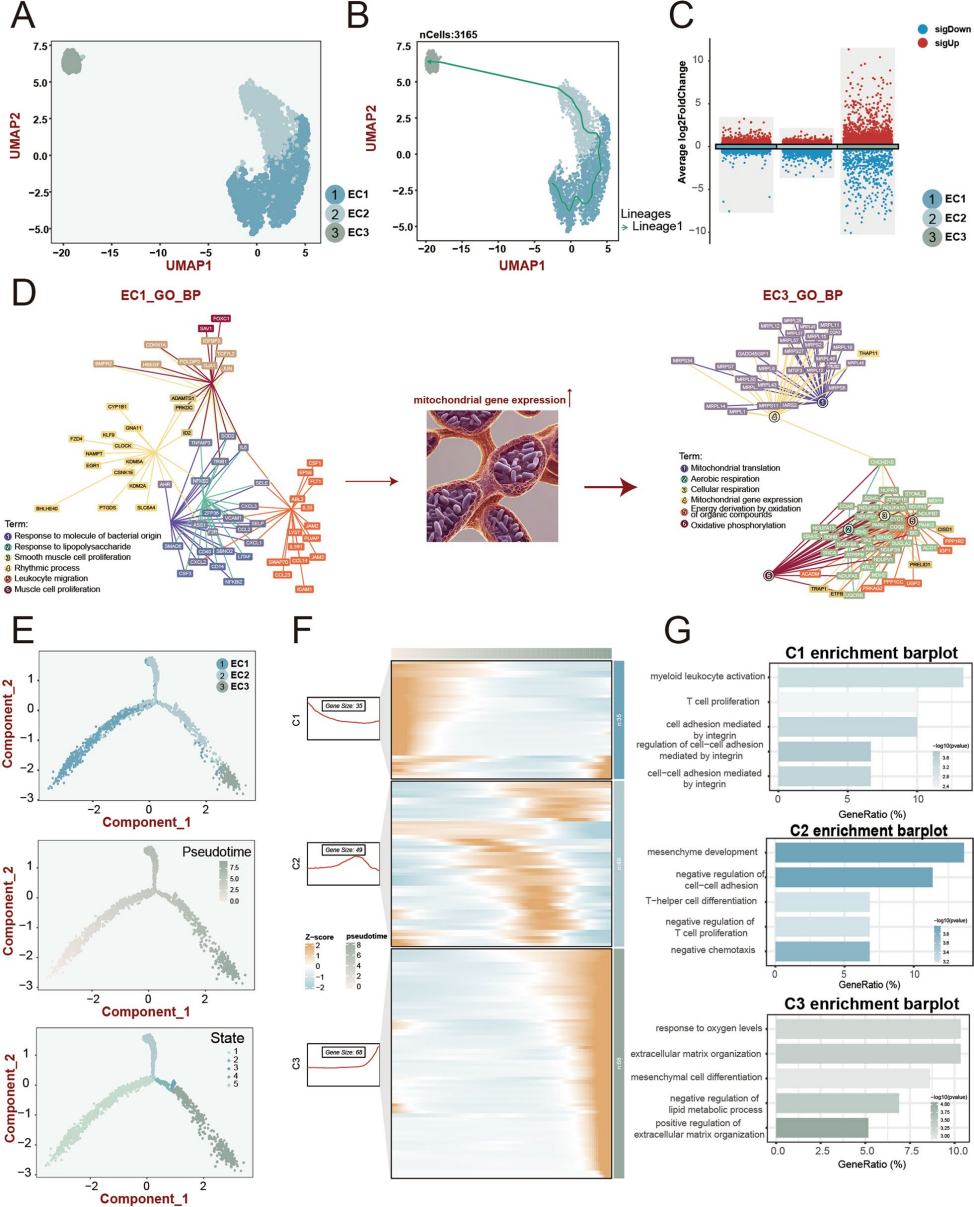
Trajectory analysis of endothelial populations.** A** UMAP plots showed that a total of 3165 cells from the PAH and control group were recruited for further trajectory analysis. **B** The developmental trajectory of endothelial cells identified by Slingshot. **C** Volcano plot exhibited the DEGs among EC1, EC2, and EC3. **D** Alterations of biological function enrichment along the developmental trajectory. **E** The cell differentiation trajectory identified by Monocle 2, coloured-coded by the associated EC subsets (top), pseudotime (middle), and states (bottom). **F** Heatmap demonstrated different expressed patterns of clustered DEGs (termed C1, C2, and C3) along the pseudotime trajectory. **G** Bar plot showed selected enriched pathways related to C1, C2, and C3

To gain a deeper understanding of the dynamics of cell differentiation, we conducted pseudotime developmental trajectory analysis. Following further dimensionality reduction and clustering, endothelial cells were categorized into three distinct populations: EC1, EC2, and EC3 (Fig. 4A). Initial Slingshot pseudotime trajectory analysis inferred the lineage structure with EC1 at the starting point position and EC3 at the end point position (Fig. 4B). We further examined subsets of ECs to elucidate the functional differentiation. Compared to EC1 and EC2, EC3 exhibited obvious heterogeneity and possessed 602 significant marker genes (Fig. 4C, Table S4). As depicted in Fig. 4D and S4B, immune-related pathways were activated during the initial stages of differentiation, including response to bacterial molecules and leukocyte migration. Subsequently, as cell differentiation progressed, there was an up-regulation in mitochondrial gene expression, followed by enrichment in mitochondrial transport and oxidative phosphorylation pathways.

**Fig. 5:**
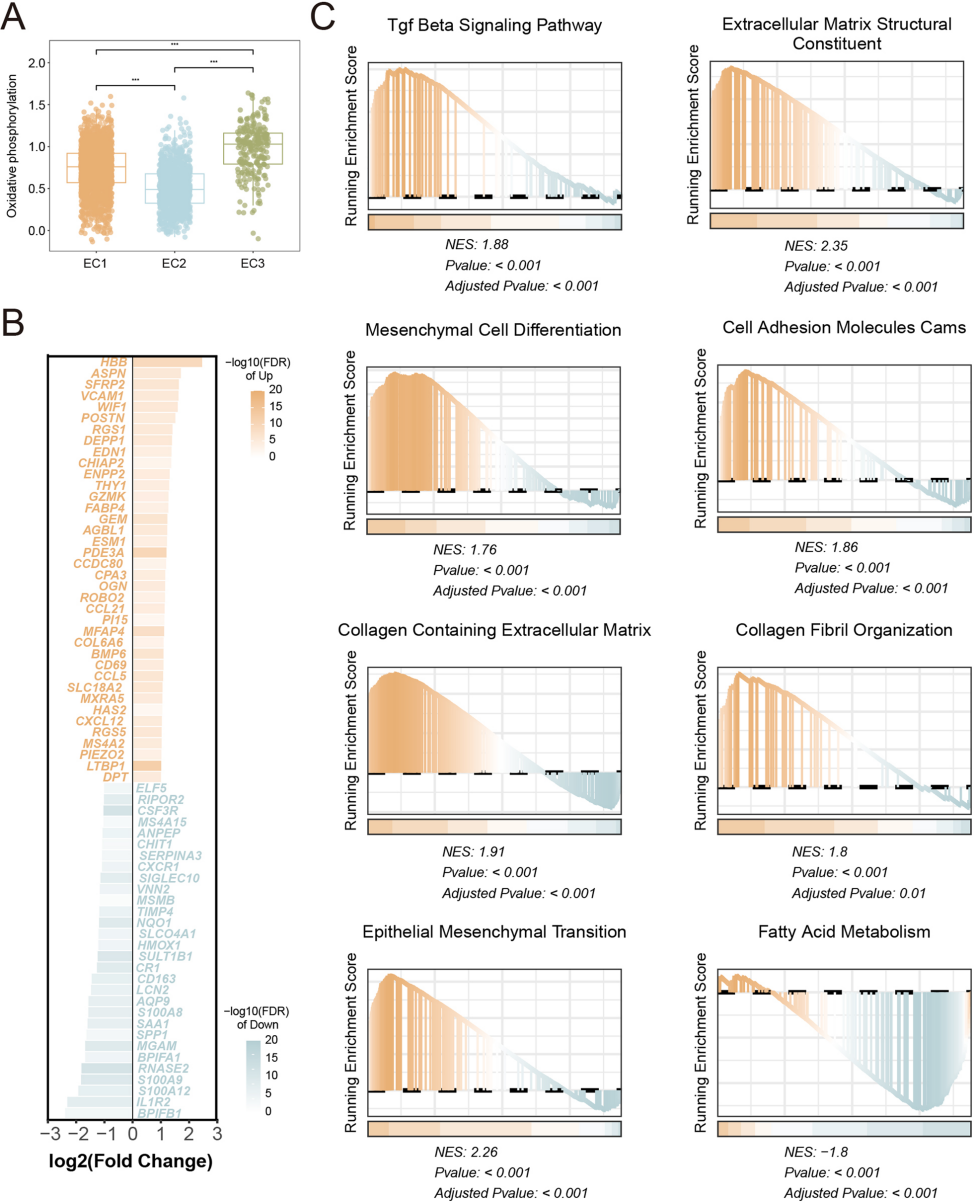
Bulk RNA-seq revealed biological characters in PAH.** A **Comparison of oxidative phosphorylation levels among three EC subsets. **B** Bar plot showed up-regulated genes and down-regulated genes in PAH. **C** GSEA estimated differences in pathway activity across PAH samples and control samples

We further reconstructed the cell differentiation trajectory using the monocle2 algorithm, and obtaining a similar pseudotime trajectory (Fig. 4E). By clustering pseudotime correlating genes, we identified three distinct gene expression patterns (Fig. 4F), namely Cluster1 (C1), Cluster2 (C2), and Cluster3 (C3). C1, which exhibited high expression at the onset of differentiation, was enriched in immune-related pathways such as myeloid leukocyte activation, T cell proliferation, and cell–cell adhesion-related pathways (Fig. 4G). C2, on the other hand, showed high expression during mid-differentiation and was associated with mesenchyme development, negative regulation of cell–cell adhesion, and negative chemotaxis pathways (Fig. 4G). Finally, C3 displayed high expression towards the end of differentiation and possessed correlation with pathways associated with mesenchymal transition, including extracellular matrix organization, cell transition, and positive regulation of extracellular matrix organization (Fig. 4G). For cell–cell communications, EC3 showed enhanced connection with fibroblasts and lacked communication with immune cells compared to EC1 (Figure S4C, D). Besides, we performed the quantitative metabolic activity analysis to validate our results, which indicated the most metabolically active in oxidative phosphorylation in EC3 (Fig. 5A, Figure S5A).

### Functional enrichment analysis in the PAH patient cohort

We performed further analysis of the lung tissue RNA-seq data from PAH patients to confirm our conclusions from the single-cell level. After analyzing differentially expressed genes, 38 up-regulated genes and 30 down-regulated genes were identified (Fig. 5B, Table S5). GSEA was conducted to evaluate the biological processes associated with the ranked differential gene expression matrix. Likewise, we observed enrichment of mesenchymal differentiation-related pathways in PAH patients, including mesenchymal cell differentiation, collagen-containing extracellular matrix, EMT, extracellular matrix structure, and collagen fibril organization (Fig. 5C). In addition, the activation of TGF-β pathway and down-regulation of fatty acid metabolism pathway matched our findings obtained at the single-cell level (Fig. 5C).

### Identification of EndMT-related modules and genes

**Fig. 6:**
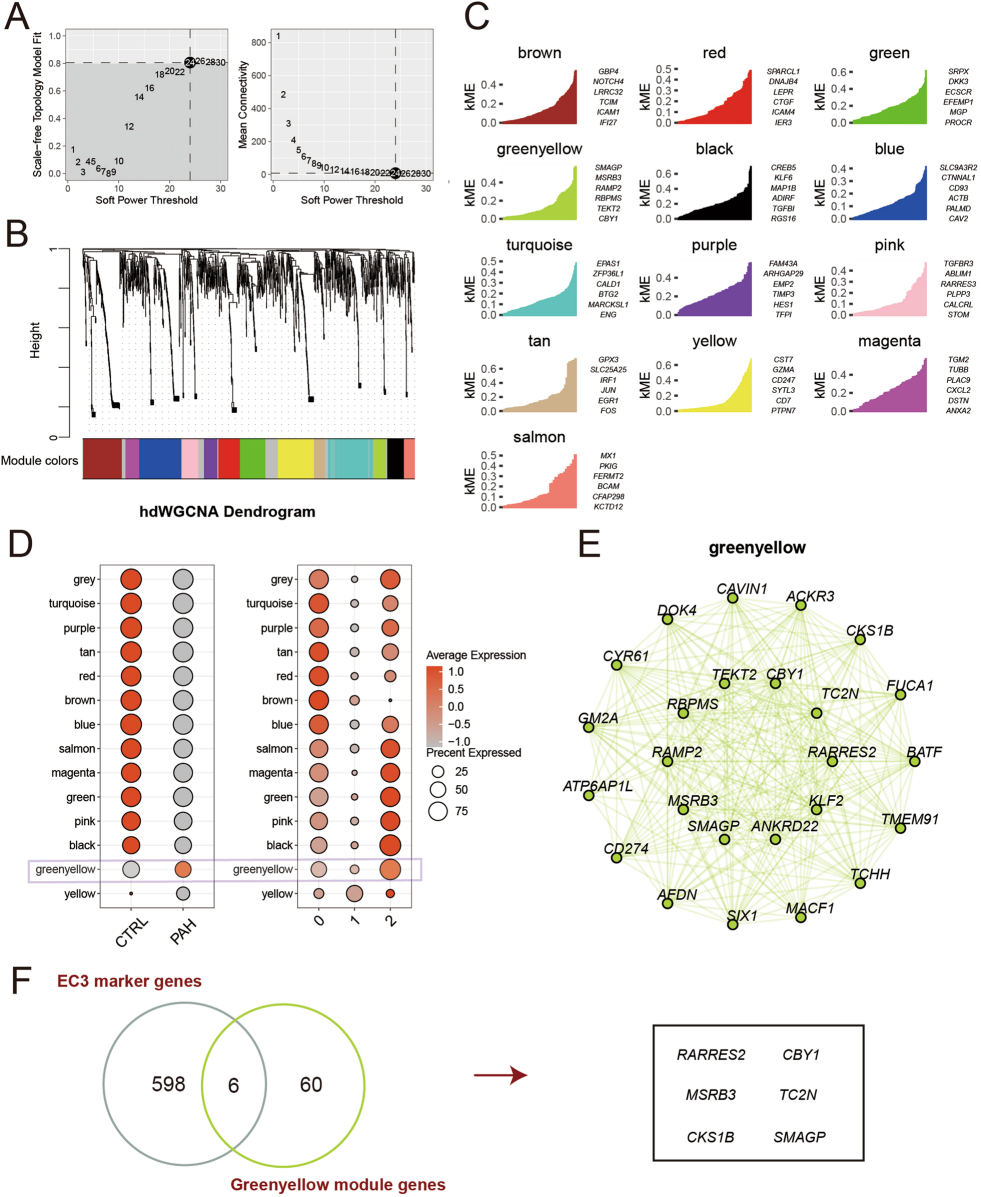
hdWGCNA identified key genes associated with EC3.** A** Scale-free topological indices at various soft-thresholding powers (left); correlation analysis between the soft-thresholding powers and mean connectivity ofthe network. Setting the soft power of β to 24, resulted in a high scale-free R2 (> 0.8), indicating a good fit to the scale-free topology model, and the stabilization ofmean connectivity (**B**) Dendrogram showed 13 modules identified from the topological overlap matrix. **C** Module eigengenes and their connectivity (kMEs) of 13 modules. **D** Heatmap showed average expression of each module between two groups and among three EC subsets. **E** Network plots showed hub genes of the greenyellow module. **F** Venn plots showed six identified EndMT pattern genes

Collectively, scRNA-seq and bulk RNA-seq analysis unveiled the presence of EndMT in PAH ECs. As described, EC3 showed the most significant connection with EndMT. Therefore, we further performed hdWGCNA analysis on EC3. The single-cell transcriptome co-expression matrix was constructed with the optimal soft threshold of 24 (fraction = 0.05) (Fig. 6A). According to the scale-free network structure, 13 modules were identified. Meanwhile, their dendrogram, module eigengenes, and module connectivity (kMEs) were demonstrated (Fig. 6B, C). Interestingly, the greenyellow module possessed a higher expression level in both EC3 and the PAH group and was recognized as the EndMT-related module (Fig. 6D, E). The intersection of 66 greenyellow module genes and 602 significant EC3 marker genes were defined as EndMT pattern genes (ETPGs), including RARRES2, CBY1, MSRB3, TC2N, CKS1B, and SMAGP (Fig. 6F, Table S6). We evaluated the ability of each gene to recognize PAH separately, with RARRES2 standing out (Figure S5B). Besides, compared to the control group, elevated mRNA expression levels of RARRES2, TC2N, CKS1B, and SMAGP were detected in human hypoxia-induced PAECs (Figure S6A). Though higher expression of CBY1 and MSRB1 were detected in human hypoxia-induced PAECs, the difference compared to the control group does not achieve statistical significance (Figure S6A).

### Machine learning-based integrative program generates PETS

**Fig. 7:**
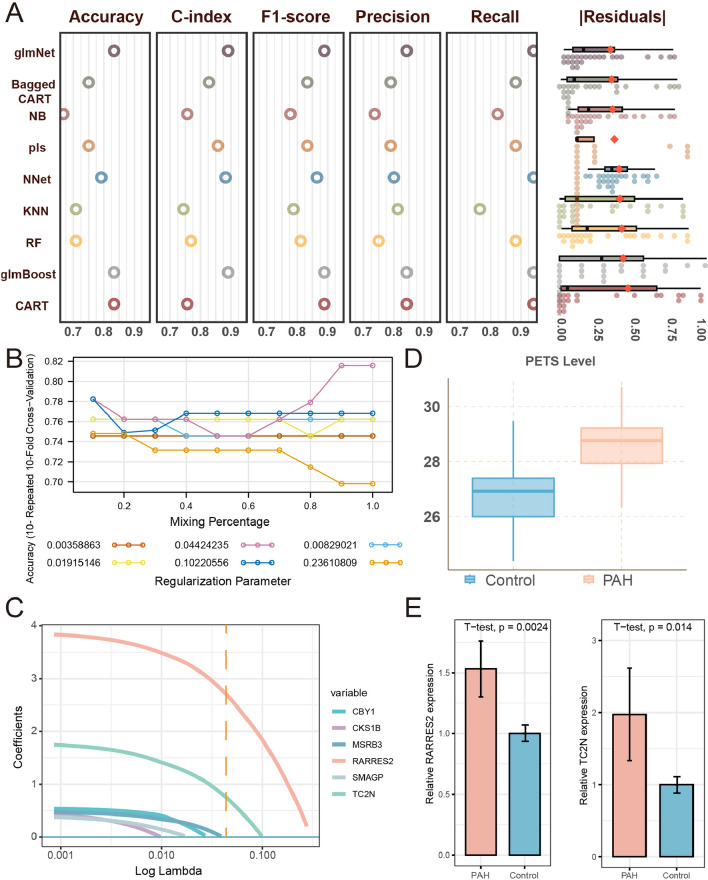
Machine learning-based integrative program generates PETS.** A** Comprehensive performances of nine types of learners. Boxplots depicted the distribution of the residuals, with red highlighted dots representing root-mean-square of residuals (RMSR). Circles showed the distribution of recall, precision, F1-score, C-index, and accuracy of each learner. **B**-**C** The determination of the optimal λ was obtained when the partial likelihood deviance reached the minimum value, and further generated glmNet coefficients of the most useful prognostic genes. **D** Boxplot showed PETS levels in PAH and control group. **E** Bar plot showed mRNA expression levels of RARRES2 and TC2N in hypoxia induced mouse PAECs and control

ETPGs were incorporated into our machine learning-based integrative program to establish PETS. We employed nine classical learners using tenfold cross-validation with 10 repetitions to fit models and evaluated their performance using five metrics, including accuracy, C-index, F1-score, precision, recall, and root-mean-square of the residuals (RMSR), in the testing cohort. The generalized linear model demonstrated superior classification capabilities based on these performance metrics (Fig. 7A, Table S7). Following the determination of optimal regularization parameters by considering accuracy variation in cross-validation, the final model was identified as follows: PETS = 2.6926723 × RARRES2 + 0.7410611 × TC2N (Fig. 7B, C). Importantly, PAH patients exhibited higher PETS levels compared to healthy individuals, and PETS levels showed a better ability to recognize PAH (Fig. 7D), suggesting the potential of PETS levels as a biomarker for PAH. Meanwhile, in mouse hypoxia-induced PAECs, elevated mRNA expression levels of RARRES2 and TC2N were detected, consistent with our conclusions (Fig. 7E). As the most prominent factor in PETS, we conducted immunofluorescence staining with the protein encoded by RARRES2 (chemerin) and the EC marker gene in hypoxic PAH mice lung paraffin sections. As illustrated in Figure S6B, chemerin was upregulated in PAH ECs.

## Discussion

PAH was a chronic, progressive and rapidly fatal disease with a poor prognosis, high mortality rate and lack of curative treatment options (Hassoun 2021). The early diagnosis and treatment of PAH have remained a significant challenge for clinicians (Vachiery and Gaine 2012; Poch and Mandel 2021). As a multifactorial disease, the development of PAH has been demonstrated to involve numerous biological processes that affect ECs, SMCs, and fibroblasts (Maron, et al. 2021). Fortunately, rapid development of high-throughput sequencing technology provided an opportunity to explore these biological processes. In recent years, researchers have completed a number of studies on PAH using this technology (Yao, et al. 2021; Tang, et al. 2022; He, et al. 2023). However, most of them focused on bulk RNA sequencing, and there is still limited research on the application of single-cell sequencing to explore the specific biological processes of PAH. In this study, we demonstrated that there were complex and heterogeneous changes in cell proportion, cell function, distributed preference, and cell–cell communication among PAH patients at the single-cell resolution. By integrating scRNA-seq and bulk RNA-seq data, we confirmed the occurrence of the EndMT process in the early stages of PAH, which was crucial for PAH development. Through further analysis of the EndMT process, we identified EndMT pattern genes (ETPGs). Based on ETPGs, our machine learning-based integrative program developed an optimal classifier (referred to as PETS), which effectively distinguished between PAH patients and healthy individuals.

The histologic features of PAH were complex and varied, but the uncontrolled growth of ECs, SMCs, and fibroblasts was a common feature (Southgate, et al. 2020). Consistently, we observed an elevated proportion of these cells and mast cells in PAH, which also exhibited preference and leading contribution to the disease. Furthermore, enhanced intercellular interactions were observed in PAH, particularly involving ECs and SMCs interacting with other cells. In recent years, ECs have gained interest, as they are affected in early PAH. For instance, Liang et al. Reported that TGF-β is a crucial mediator of EndMT (Liang, et al. 2022). Wang et al. Found a low level of mediator complex subunit 1 (MED1) in PAH patients’ lung tissue and endothelial cells, which promoted vascular remodeling via the TGF-β signaling pathway (Wang, et al. 2022). Notably, ECs from PAH patients revealed significant biological processes related to alterations in cell proliferation and EndMT, including oxidative phosphorylation, myogenesis, and the TGF-β signaling pathway. TGF-β signaling pathway activation, mesenchymal cells production and differentiation were detected in the bulk RNA-seq dataset as well. Moreover, ECs showed lower incoming interactions and higher outgoing interactions in the PAH group, which might indicate functional dysfunction.

These rousing findings prompted us to conduct further research on ECs. Surprisingly, we identified a unique unreported subgroup, which possessed heterogeneity and located at the terminal differentiation stage (termed EC3). At the gene expression level, it displayed significant differences compared to other ECs, mainly pointing to elevated oxidative phosphorylation activity. Subsequently, EC3 demonstrated mesenchymal differentiation and down-regulation of lipid metabolism gene expression patterns in the differentiation fate. Consistent findings occurred in the PAH lung tissue RNA-seq cohort, which refined the understanding of EC3. Of note, EC3 displayed enhanced cell–cell communications with fibroblasts. EndMT has the potential to induce the transformation of endothelial cells into smooth muscle-like cells with enhanced abilities in proliferation and migration, thereby directly contributing to the development of PAH or indirectly through paracrine effects on vascular intimal and medial proliferation (Suzuki, et al. 2018; Gorelova, et al. 2021). Therefore, it was of consequence to identify early diagnosis and treatment targets based on EC3.

At the single-cell level, we further constructed the topological overlap matrix and established the co-expression network to determine meaningful modules and feature genes. Based on the EC3-specific module and EC3 marker genes, we identified six EndMT pattern genes (ETPGs), including RARRES2, CBY1, MSRB3, TC2N, CKS1B, and SMAGP. Then, we aimed to develop an EndMT-related signature for the diagnosis and treatment of PAH patients. Nowadays, machine learning is emerging as a robust method for medical decision-making, demonstrating superior performance compared to traditional models in disease diagnosis, prognosis assessment, and disease progression prediction (Liu, et al. 2022; Silva, et al. 2022; Sun, et al. 2022). However, predictive power deficiency caused by researchers’ selection bias and knowledge limitations hindered the clinical application of many biomarkers (Shimron, et al. 2022; Volovici, et al. 2022). In contrast, we developed a systematic framework to fully exploit nine machine learning algorithms and performed comprehensive evaluations. Finally, the glmNet algorithm provided the optimal PETS, which exhibited powerful predictive performance. Therefore, we believe PETS might serve as a key biomarker of PAH diagnosis and a target for anti-EndMT therapy.

Retinoic acid receptor responder 2 (RARRES2), also known as chemerin, is an important adipokine in obesity, inflammation, and cardiovascular disease. Previous studies have reported that chemerin could promote angiogenesis and ROS production and decrease insulin signaling and nitric oxide production in vascular endothelial cells (Bozaoglu, et al. 2010; Neves, et al. 2018; Ferland, et al. 2020). Recently, serum chemerin levels have received increasing attention from clinical researchers, including cardiovascular and renal disease-related evaluations (Bonomini and Pandolfi 2016; Zhou, et al. 2019; Szpakowicz, et al. 2021). Relying on serum chemerin and TC2N levels, our PETS was a potential tool to indicate the risk of PAH at an early stage in clinical practice, which was conducive for early intervention and timely treatment. Additionally, new therapeutic drugs targeting chemerin and related signaling proteins, such as the receptor chemokine-like receptor 1 (CMKLR1), are also in progress. A CMKLR1 inhibitor, CCX832, has been extensively studied in vitro and in vivo, exhibiting significant ability to ameliorate chemerin-induced vascular dysfunction (Xie and Liu 2022). Thus, CCX832 may be a treatment option for PAH patients, but only after rigorous preclinical studies.

The following limitations in this study should be recognized: (1) Procedures bias from the system biology potentially cannot fully recapitulate the overall disease exacerbation. (2) All samples were retrospective data in our current research, a prospective study should be performed. (3) Cell dissociation-associated spatial information deficiency influenced the prediction of cell communication, further single-cell multi-omics studies would further expound the pathogenesis of PAH. (4) Since the current study is based on a high-throughput RNA data, multi-omics’ comprehensive studies would be crucial addition.

## Conclusion

In conclusion, we further depicted the biological landscape in PAH by incorporating both scRNA-seq and bulk RNA-seq data. Using a computational biology approach, we explored unique alterations in ECs and developed a robust signature (PETS) for PAH diagnosis. These findings offered clues for future biological and clinical research and should prove valuable in prospective therapy strategies, specifically targeting EndMT.

## Supplementary Information

Additional file 1: Figure S1: The distribution of gene numbers of cells before and after quality control.

Additional file 2: Figure S2: (A) UMAP plot showed 21 identified cell clusters. (B) Dot plots of canonical marker genes.

Additional file 3: Figure S3: Boxplots depicted the percentage of the distinct cell types between PAH and control groups.

Additional file 4: Figure S4: (A) Circle plot displayed the number of interactions. Red lines indicated increased communication in PAH compared with control group, while blue lines represented decreased communication. The line thickness was proportional to unique ligand-receptor interactions. Autocrine circuits were symbolized by loops. (B) Enriched pathways for three EC subsets. (C-D) Cell-cell communications analysis for EC1 and EC3.

Additional file 5: Figure S5: (A) Metabolism activity for three EC subsets. (B) Respective ROC curve for PAH distinguishment of ETPGs.

Additional file 6: Figure S6: (A) Barplot showed mRNA expression levels of RARRES2, TC2N, CBY1, CKS1B, MSRB3, and SMAGP in hypoxia-induced human PAECs and control. (B) Hypoxic PAH mice lung paraffin sections were stained with chemerin (green) and CD31 (red), and were observed by immunofluorescence microscopy. DAPI staining was used to visualize the nuclear areas. The scale bar represents 20 μm.

Additional file 7: Table S1: Top differentially expressed genes between PAH and control groups.

Additional file 8: Table S2: Contribution scores of different cell types.

Additional file 9: Table S3: Differentially enriched pathways in distinct cell types between PAH and control groups.

Additional file 10: Table S4: Significant EC3 marker genes.

Additional file 11: Table S5: Identified PAH differentially expressed genes from the bulk RNA-seq cohort.

Additional file 12: Table S6: Greenyellow module genes.

Additional file 13: Table S7: Evaluation of multiple machine learning models.

## Data Availability

No datasets were generated or analysed during the current study.

## Abbreviations

PAH: Pulmonary arterial hypertension
EC: Endothelial cell
EndMT: Endothelial-to-mesenchymal transition
scRNA-seq: Single-cell RNA sequencing
ETPG: EndMT pattern gene
DEG: Differentially expressed gene
HVG: Highly variable gene
UMAP: Uniform manifold approximation and projection
KEGG: Kyoto encyclopedia of genes and genomes
GO: Gene ontology
GSEA: Gene set enrichment analysis
EMT: Epithelial mesenchymal transition
RMSR: Root mean square of the residuals
SMC: Smooth muscle cell

## Code availability

The code that supports the findings of this study is available from the corresponding author upon reasonable request.
