# Supplementary figures and images for: Assessing personalized molecular portraits underlying endothelial-to-mesenchymal transition within pulmonary arterial hypertension
Source: Mol Med. 2024 Oct 26;30:189. doi: 10.1186/s10020-024-00963-z (PMC11513636; doi:10.1186/s10020-024-00963-z)

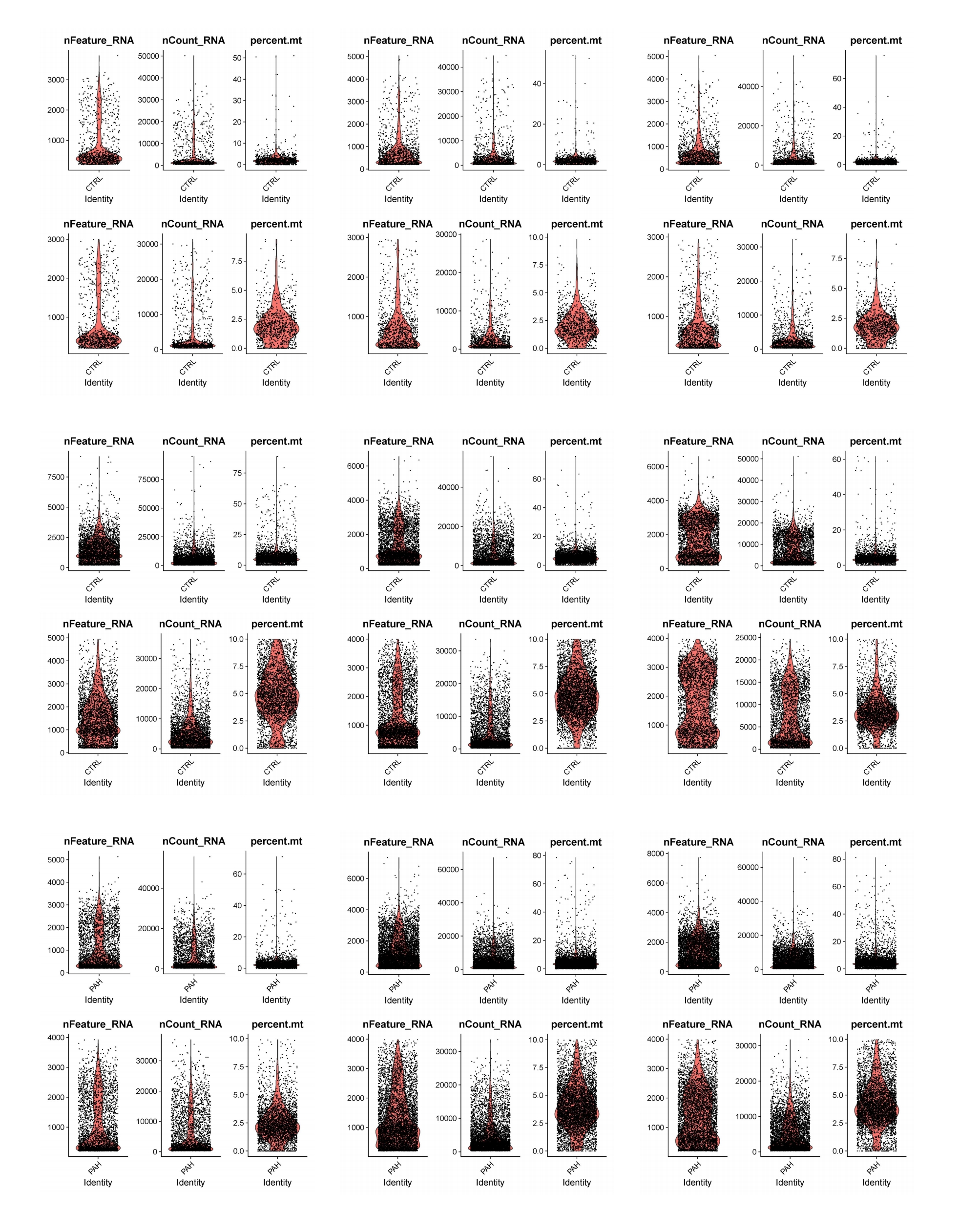

Supplement: Supplementary file 1 — Additional file 1: Figure S1: The distribution of gene numbers of cells before and after quality control. [file 10020_2024_963_MOESM1_ESM.jpg]

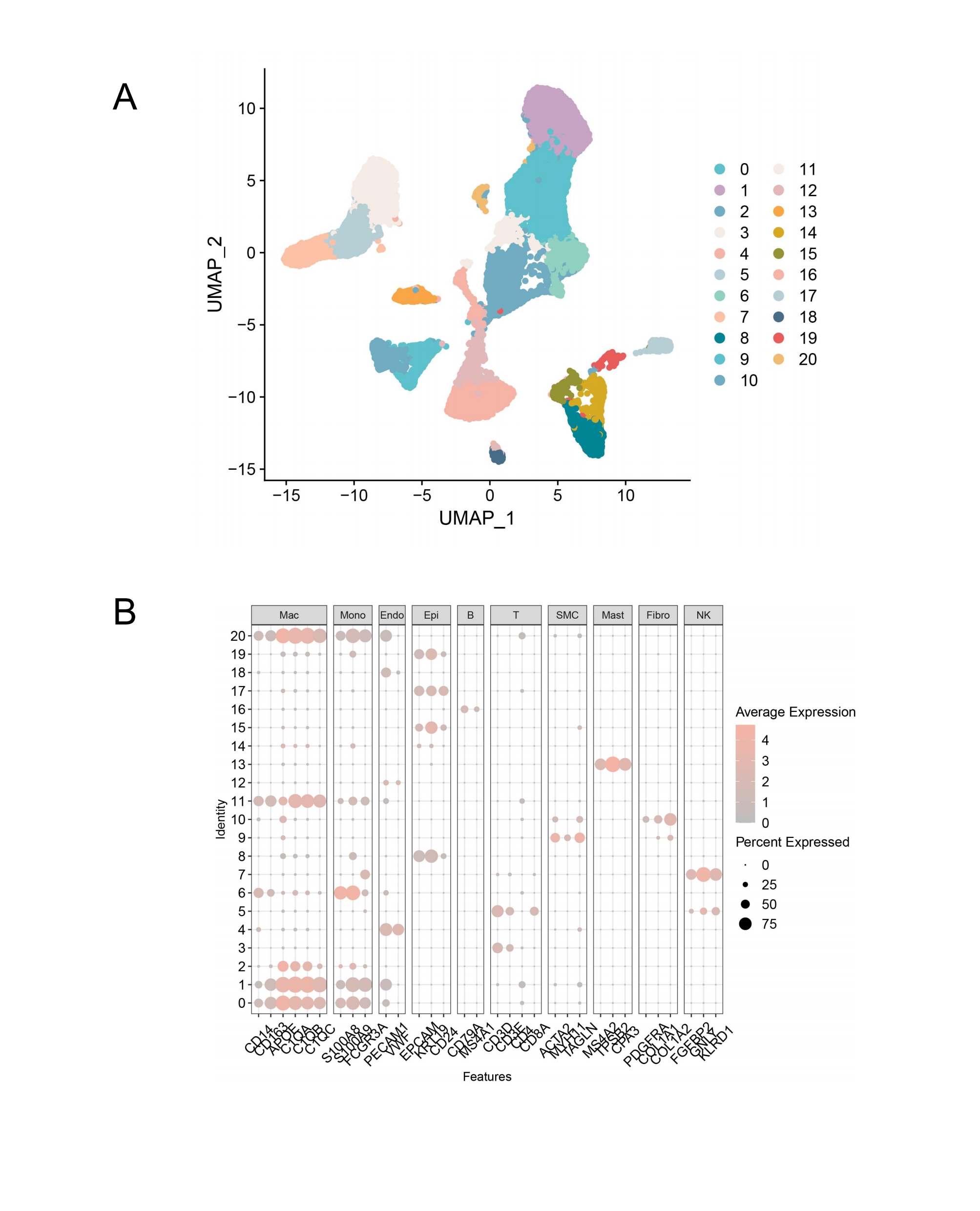

Supplement: Supplementary file 2 — Additional file 2: Figure S2: (A) UMAP plot showed 21 identified cell clusters. (B) Dot plots of canonical marker genes. [file 10020_2024_963_MOESM2_ESM.jpg]

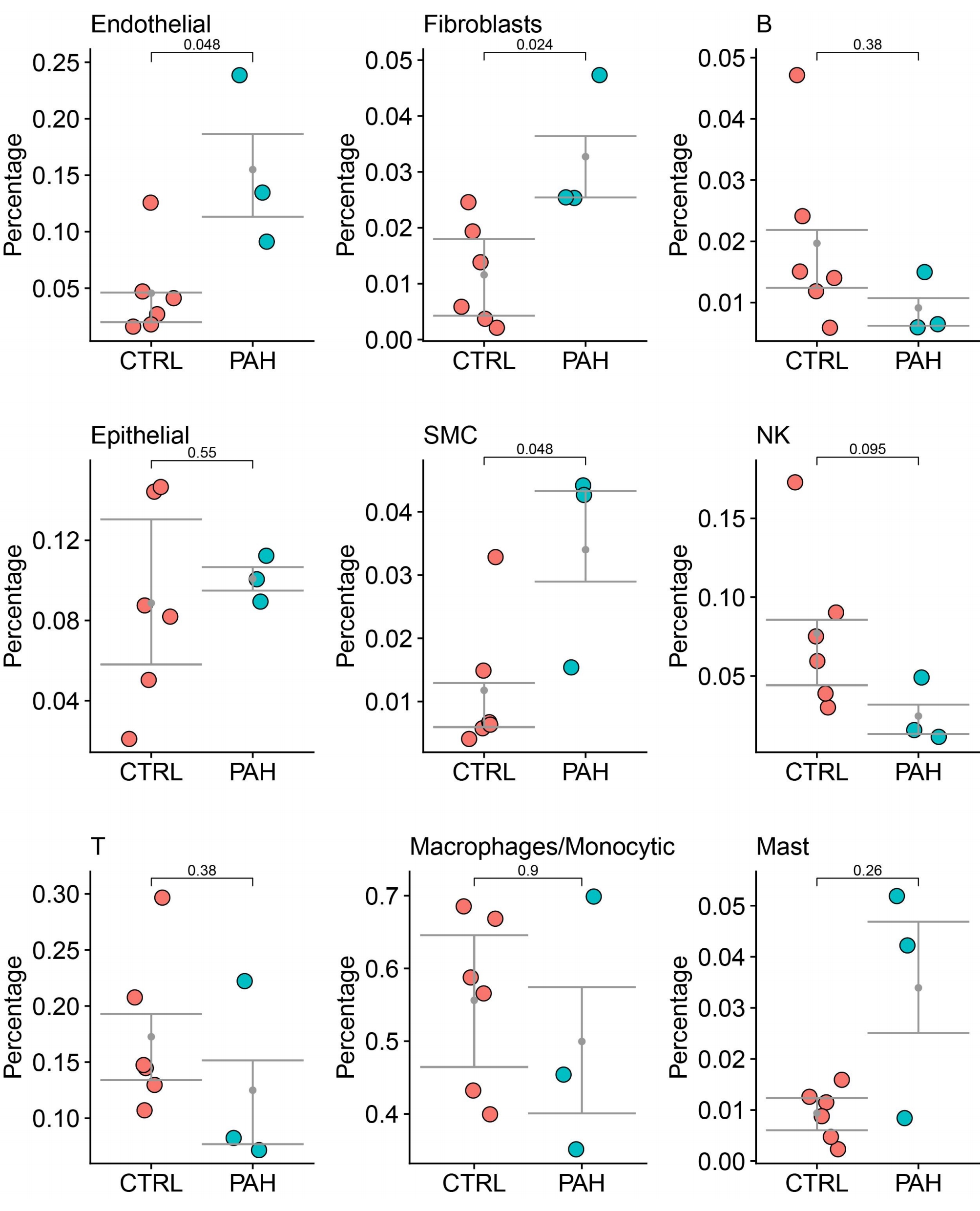

Supplement: Supplementary file 3 — Additional file 3: Figure S3: Boxplots depicted the percentage of the distinct cell types between PAH and control groups. [file 10020_2024_963_MOESM3_ESM.jpg]

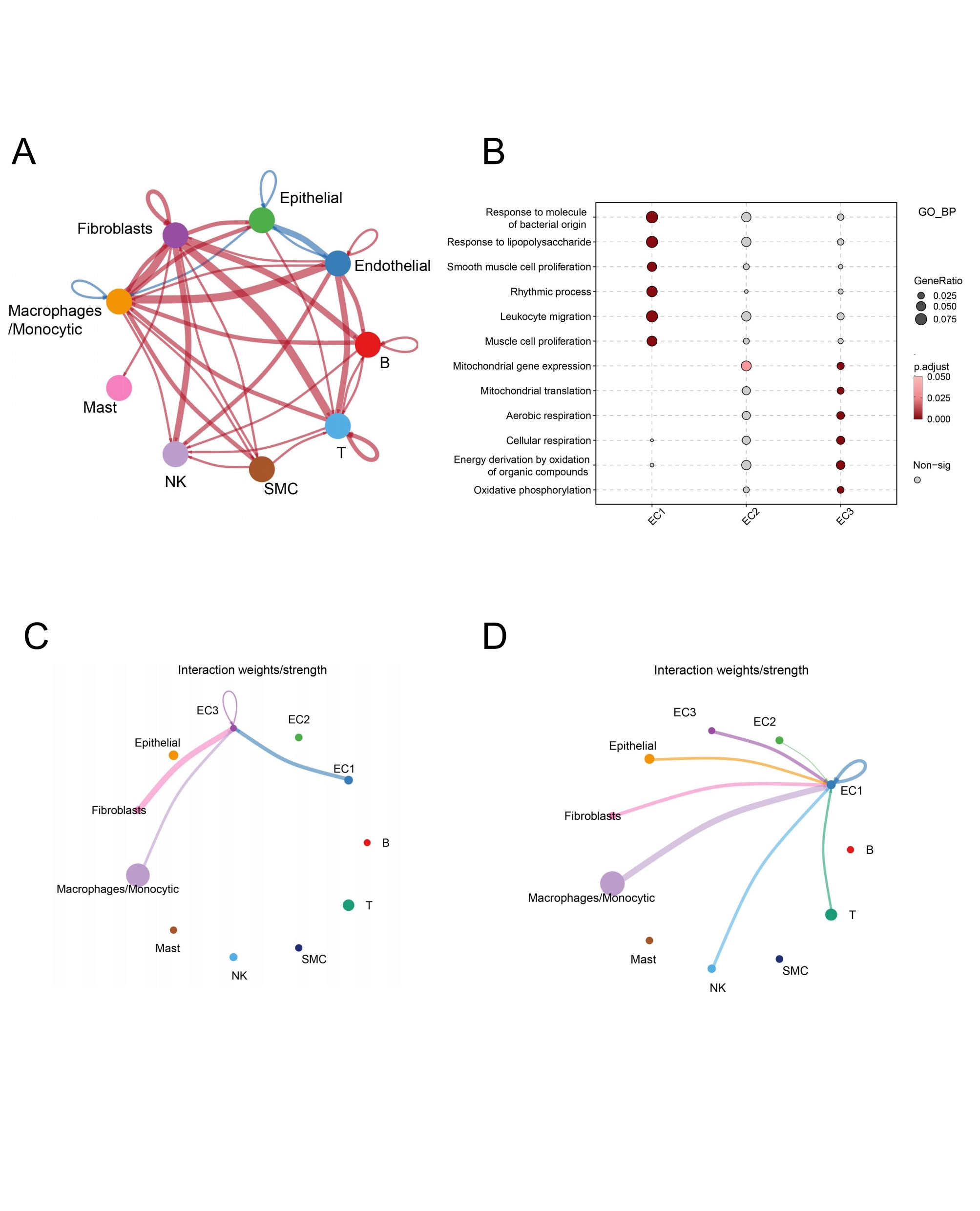

Supplement: Supplementary file 4 — Additional file 4: Figure S4: (A) Circle plot displayed the number of interactions. Red lines indicated increased communication in PAH compared with control group, while blue lines represented decreased communication. The line thickness was proportional to unique ligand-receptor interactions. Autocrine circuits were symbolized by loops. (B) Enriched pathways for three EC subsets. (C-D) Cell-cell communications analysis for EC1 and EC3. [file 10020_2024_963_MOESM4_ESM.jpg]

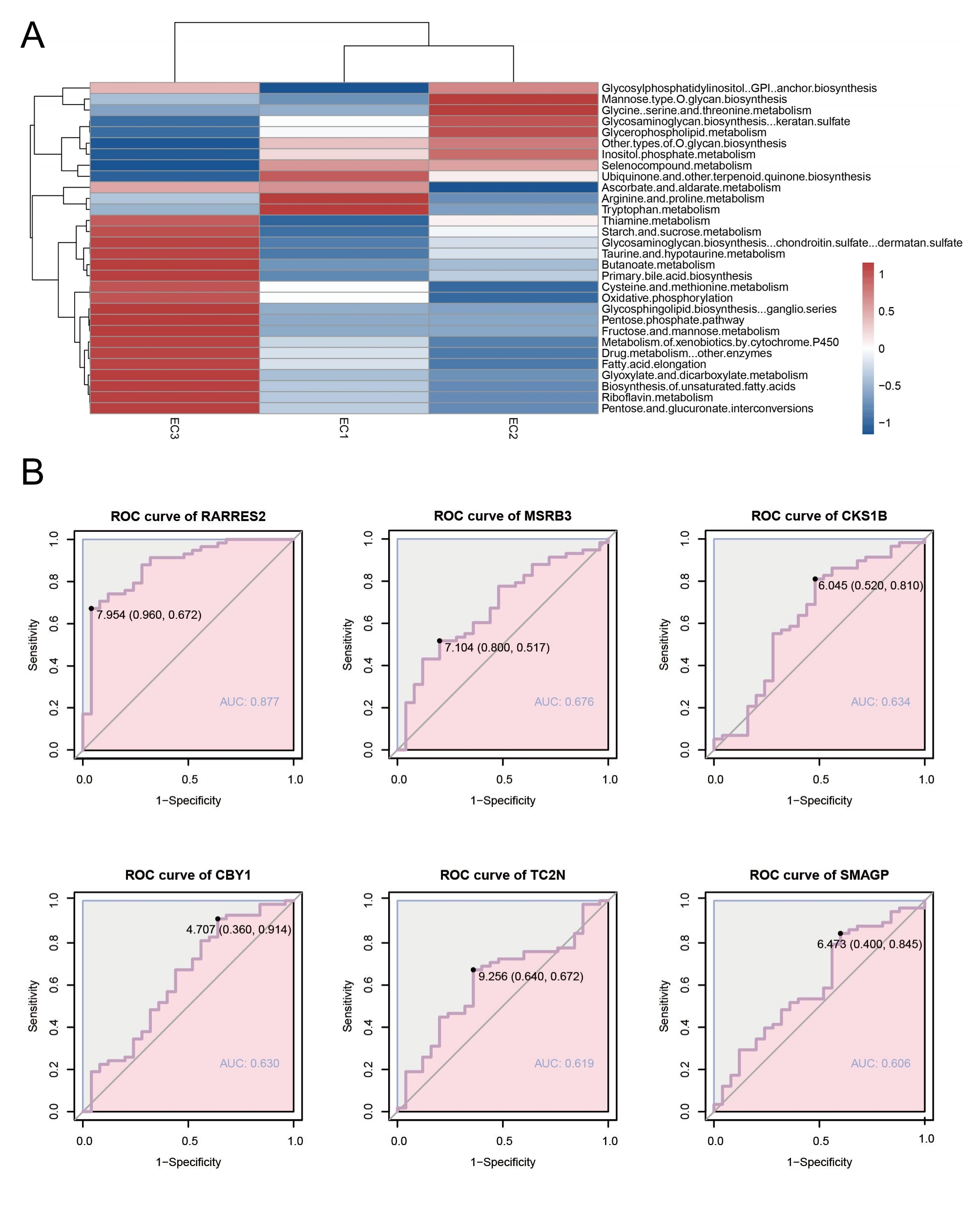

Supplement: Supplementary file 5 — Additional file 5: Figure S5: (A) Metabolism activity for three EC subsets. (B) Respective ROC curve for PAH distinguishment of ETPGs. [file 10020_2024_963_MOESM5_ESM.jpg]

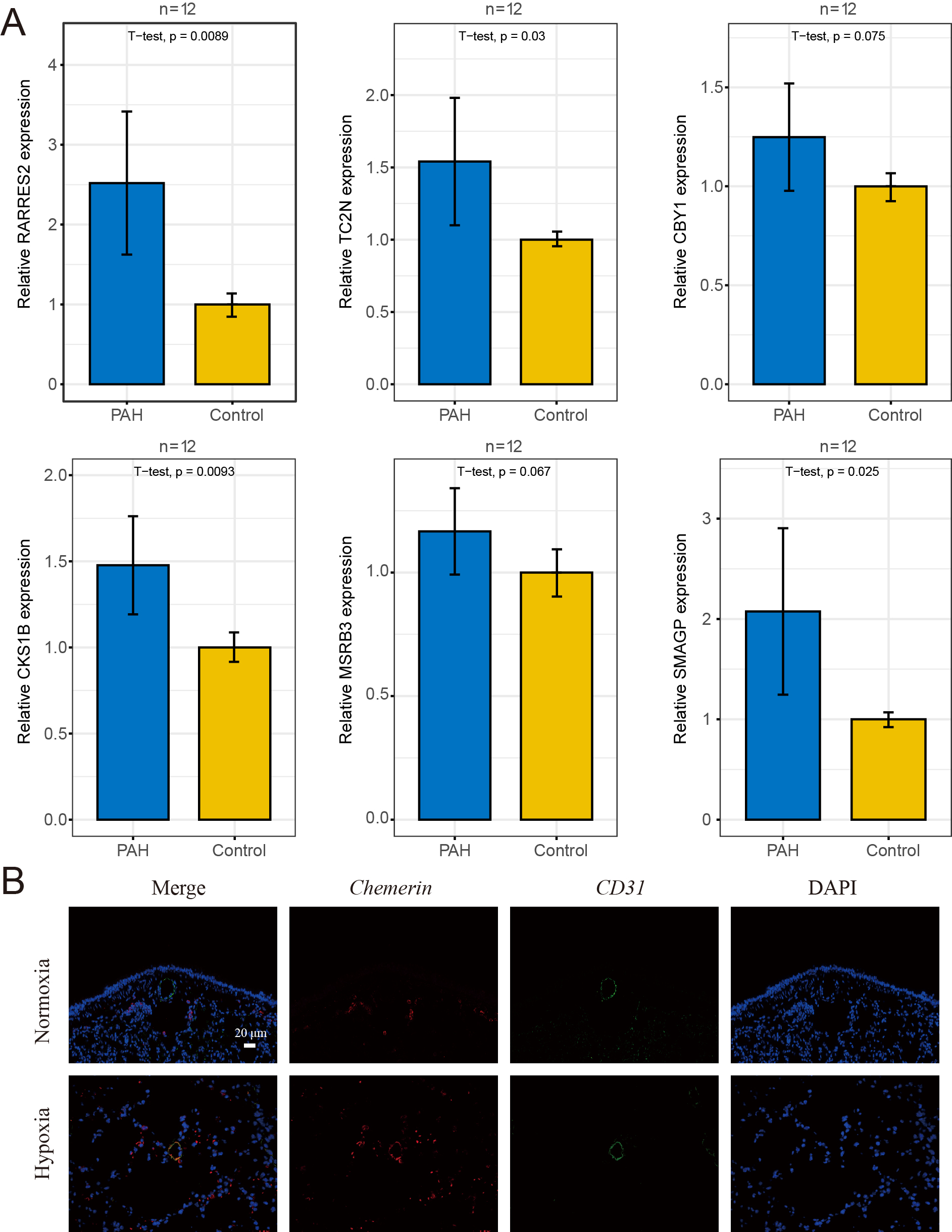

Supplement: Supplementary file 6 — Additional file 6: Figure S6: (A) Barplot showed mRNA expression levels of RARRES2, TC2N, CBY1, CKS1B, MSRB3, and SMAGP in hypoxia-induced human PAECs and control. (B) Hypoxic PAH mice lung paraffin sections were stained with chemerin (green) and CD31 (red), and were observed by immunofluorescence microscopy. DAPI staining was used to visualize the nuclear areas. The scale bar represents 20 μm. [file 10020_2024_963_MOESM6_ESM.jpg]
